# Cytotoxicity and Immunogenicity Evaluation of Synthetic Cell-penetrating Peptides for Methotrexate Delivery

**DOI:** 10.22037/ijpr.2021.114429.14842

**Published:** 2021

**Authors:** Parvin Zakeri-Milani, Saeedeh Najafi-Hajivar, Muhammad Sarfraz, Ali Nokhodchi, Hamed Mohammadi, Soheila Montazersaheb, Mehri Niazi, Maryam Hemmatzadeh, Mehdi Soleymani-Goloujeh, Behzad Baradaran, Javid Shahbazi Mojarrad, Masoud Farshbaf, Tooba Gholikhani, Hadi Valizadeh

**Affiliations:** a *Liver and Gastrointestinal Diseases Research Center and Faculty of Pharmacy, Tabriz University of Medical Sciences, Tabriz, Iran. *; b *Student Research Committee, and Department of Medical Nanotechnology, Faculty of Advanced Medical Sciences, Tabriz University of Medical Sciences, Tabriz, Iran. *; c *College of Pharmacy, Al Ain University, Al Ain 64141, UAE. *; d *Pharmaceutics Research Laboratory, School of Life Sciences, University of Sussex, Falmer, Brighton BN1 9QJ, United Kingdom. *; e *Immunology Research Center, and Department of Immunology, School of Medicine, Tabriz University of Medical Sciences, Tabriz, Iran. *; f *Stem Cell Research Center, and Faculty of Pharmacy, Tabriz University of Medical Sciences, Tabriz, Iran. *; g *Biotechnology Research Center, and Faculty of Pharmacy, Tabriz University of Medical Sciences, Tabriz, Iran. *; h *Drug Applied Research Center and Faculty of Pharmacy, Tabriz University of Medical Sciences, Tabriz, Iran.*

**Keywords:** CPPs, Drug delivery, Nanoparticles, Cancer, Immune system

## Abstract

Methotrexate (MTX) is one of the most effective therapeutics to treat different types of solid tumors; however, it suffers low permeability limiting its bioavailability and cellular uptake. To tackle this, we aim to design and fabricate different types of cell-penetrating peptides (CPPs) to improve the intracellular uptake of MTX without causing any immunogenic response. CPPs were synthesized by the solid-phase peptide synthesis method. Peptide-MTX conjugates were prepared *via* covalent binding of peptide and drug molecule. CPPs and peptide-E_8_ nanoparticles were characterized using zeta-sizer and scanning electron microscopy. Cytotoxicity of CPPs and peptide-MTX conjugates was evaluated by MTT assay. An enzyme-linked immunosorbent assay was employed to assess the IL-6 and TNF-α cytokine release profile. Amongst all sequences, W_4_R_4_-MTX possessed the highest loading efficiency (97%) and drug to peptide percentage (24.02%). The lowest loading efficiency (36%) and drug to peptide percentage (8.76%) were seen for NGRWK-MTX conjugates. The NGRWR peptide and NGRWR-E_8_ nanoparticles had acceptable size (~100 nm) with spherical and rod-like structures, respectively. The selected CPPs and peptide-MTX conjugates did not show any cytotoxicity or immunogenicity. The fabricated peptides are represented as promising carriers to improve the intracellular delivery of MTX to cancer cells with low immunogenic and cytotoxic effects on normal cells.

## Introduction

Intracellular targeting has always been a challenge in drug delivery systems designing (1). Peptide-based delivery systems have been an efficient strategy that transport the desired macromolecules across the cell membrane with low cytotoxicity and high efficiency (2, 3). Considering their nano-scale size (7 to 30 amino acids in length), cationic or amphipathic peptides offer excellent potential to be employed as an efficient carriers for small chemotherapeutics to overcome multi-drug resistance (4-10). TAT and Antp are well-known cell-penetrating peptides (CPPs) derived from human immunodeficiency virus-1 (HIV-1) and the transcription factor of Drosophila melanogaster, respectively (11-13). Chimeric peptides, such as transportan 10 (TP10), are categorized as the second type of well-known peptides (14), and the third type includes the synthetic peptides family, of which polyarginine peptides have been mostly investigated (15, 16). Lately, synthetic peptides with tailorable sequences have gained much attention towards drug delivery systems, as they implement the natural CPPs functions. Amongst the investigated CPPs in experimental models, amphipathic peptides with hydrophobic and charged residues exhibit more potential as an appropriate drug carrier (17, 18). Moreover, amphipathic CPPs appear to be more effective in delivering small molecules due to their high biocompatibility and bioactivity in biological systems (12, 19, 20). However, there might be a few immune responses developed during the treatment that can neutralize the clinical effects of therapeutic peptides or be associated with destructive immune impacts (21, 22). As the immune system plays the primary protective role of the body against all foreign toxic and pathogenic particles, it can pose a significant challenge upon the entry of novel therapeutic carriers. Interactions between new therapeutics and subsets of the immune system induce cell signaling cascades, which may consequently induce the immune factors and immunomodulation (23). The immunogenicity of the CPPs can be affected by their different physicochemical properties such as size, surface charge, amino acids sequence, hydrophilicity, morphology, and the type of conjugated cargo (24). Based on recent reports, the modified CPPs have shown no immediate immune responses (25, 26). Moschos *et al*. has evaluated the p38 siRNA delivery using Penetratin and HIV-TAT to the murine lung cells. They could show that Penetratin complexed with siRNA induced an innate immune response, whereas siRNA complexed with HIV-TAT, or Penetratin alone, did not show any immunogenic behavior (27). Therefore, designing efficient drug delivery vectors to transduce the therapeutic agents through the cell membrane is the ultimate goal; however, their possible cytotoxicity and immunogenic response induction and the resulted undesired side effects should be considered as their severe limitations. In the present study, we design and fabricate several synthetic amphipathic peptides and their conjugations with a chemotherapy drug, Methotrexate (MTX), and evaluate their *in-vitro* concentration- and time-dependent cytotoxicity and undesired immunogenic effects on the Raji cell line.

## Experimental

*Materials and methods*

*Materials*

Fmoc-Arg(Pbf)-OH, Fmoc-Trp(Boc)-OH, Fmoc-Glu(OtBu)-OH, Fmoc-Lys(Boc)-OH, Fmoc-Asn(Trt)-OH, and Fmoc-Gly-OH were obtained from AAPPTec (Louisville, KY). The coupling agents including O-(benzotriazole-1-yl)-N, N, N′, N′-tetramethyluronium tetrafluoroborate (TBTU), Hydroxy/benzotriazole (HoBt) and N, N-Diisopropylethylamine (DIPEA), scavengers (ethanedithiol, phenol, and triisopropylsilane (TIS)), and cleavage reagents (piperidine and trifluoroacetic acid (TFA)) were purchased from Sigma (St. Louis, MO). 3-(4,5-dimethylthiazol-2-yl)-2,5-diphenyltetrazolium bromide (MTT), RPMI 1640, fetal bovine serum (FBS), trypsin, ethylenediaminetetraacetic acid (EDTA), and penicillin/streptomycin were purchased from Invitrogen (Carlsbad, CA). Raji cell line was obtained from the Pasteur Institute, Tehran, Iran. All other organic reagents were of analytical grade and purchased from Sigma-Aldrich (St. Louis, MO, USA). 

*Peptide synthesis*

Eight linear peptides, including W_4_K_4_, W_4_R_4_, [WK]_4_, [WR]_4_, WRNGRWR, WRNGR, NGRWR, and [R]_6_ were synthesized by solid-phase synthesis (SPS) method on 2-chlorotrityl chloride (CTC) resin in a stepwise manner (2). Fmoc-L-amino acids were coupled to the resin in the presence of coupling reagents using TBTU (2 equivalent), HoBt (2 equivalent), and DIPEA (2 equivalent) in N, N-dimethylformamide (DMF) after mixing for 2 h, followed by resin swelling. The Fmoc deprotection process was carried out by piperidine in DMF (20% v/v). After the sequence completion, the side chain deprotection and cleavage step were carried out using TFA/TIS/phenol/distilled water (88:2:5:5, v/v/v/v) for 2 h. In the end, the peptide-coupled resins were filtered, and the solvent was evaporated to obtain dry crude peptides.

*Peptide-MTX conjugation*

Regarding the carboxylic acid end groups of MTX molecules, the peptide-MTX conjugation process was similar to that explained for peptide synthesis (28). Briefly, the obtained resin-coupled CPPs were conjugated to MTX (3 equivalent to the amount of resin), using DIPEA (2.5 equivalent), HoBt (2.5 equivalent), and TBTU (2.5 equivalent) to form peptide-MTX conjugates after 48 h under stirring. Then, the reaction completion was confirmed by the Kaiser test. Finally, the peptide-MTX conjugates were cleaved from the resin using TFA/TIS/phenol/distilled water (88:2:5:5, v/v/v/v) 2 h, precipitated in cold diethyl ether, and washed three times. The supernatant solution was analyzed at 365 nm using an ultraviolet-visible (UV-Vis) spectrophotometer (Shimadzu, Japan) to calculate the MTX loading efficiency according to the equation below:

$\mathrm{Drug loading efficiency\%}=\left[\frac{\mathrm{amount of loaded drug in mg}}{\mathrm{amount of added drug in mg}}\right]\times 100$

*Synthesis of peptide-E*_8_ *nanoparticles*

First, 2.5 mg of the selected peptide (NGRWR, [WK]_4_ or [WK]_4_-MTX) was dissolved in 50 µL dimethyl sulfoxide (DMSO). Then poly-glutamate (E_8_) was added in a 1:5 ratio (peptide: E_8_) under ultrasonication for 2 h to form a salt bridge between peptide and E_8_ and create the nanoparticles. The obtained solution was filtered through a 0.2 μm syringe filter and lyophilized.

*Characterization*

The size and morphology of NGRWR peptide and NGRWR-E_8 _nanoparticles (as representatives) were examined by scanning electron microscopy (SEM, Mira3 FEG-SEM Tescan 5.0 kV). Samples were prepared by drop-casting of a 5 mM aqueous solution (20 µL) onto the mica surface. Then, they were lyophilized, coated with gold, and analyzed with a high volume mode of SEM. Zetasizer Nano ZS (Malvern Instruments, Worcestershire, UK) was employed to examine the mean hydrodynamic diameter, size distribution, and zeta potential of NGRWR peptide and NGRWR-E_8_ nanoparticles. To this end, the samples were diluted in distilled water (pH 7.4), and the experiment was repeated three times per sample at 25 °C.

*Cell culture and MTT assay*

Human lymphoma cell line (Raji) was cultured in 75 cm^2^ cell culture flasks containing RPMI 1640 medium (Gibco), which was supplemented with 10% FBS, 2 mM L-glutamine, 100 units/mL penicillin, and 100 µg/mL streptomycin (29). The cell culture was carried out in a humidified atmosphere of 5% CO_2_, 95% air, and 37 °C. The cell viability of CPPs and peptide-MTX conjugates was determined by MTT assay in Raji cells. Hence, 4 × 10^4^ Raji cells/well were seeded into a 96-well plate and cultured in the condition mentioned above for 24 h. Then, cells were treated with different CPPs including W_4_K_4_, W_4_R_4_, [WK]_4_, [WR]_4_, WRNGRWR, WRNGR, NGRWR, and [R]_6 _(5, 10, 25 and 50 mM) and peptide-MTX conjugates (25 and 50 nM of MTX) for 24, 48 and 72 h. The cell viability was determined by measuring the amount of MTT transformed to formazan salt using an ELISA reader at 570 nm (Bioteck Instruments, Winooski, VT, USA). The results were the means of three independent experiments performed in duplicate.

*Immunosorbent assay*

Cells were incubated with selected CPPs (5 µM), their conjugations with MTX (25 nM of MTX), [WK]_4_-E_8 _nanoparticles (5 µM), [WK]_4_-MTX-E_8_ nanoparticles (25 nM of MTX), and free MTX (25 nM) for 48 h. As a positive control, cells were incubated with 10 μg/mL of lipopolysaccharide (LPS) to induce IL-6 and TNF-α release. Then, the supernatant was collected and IL-6 and TNF-α secretion was evaluated using standard ELISA kits.

*Statistical analysis*

Statistical analysis was performed by GraphPad Prism v6.07 (Graph Pad Software, CA, USA). In the cytotoxicity and immunogenicity studies, one-way ANOVA was employed, followed by a multi-comparison test, where *p* < 0.05 was quoted as significance. 

## Results

*Synthesis and characterization*

**Figure 1:**
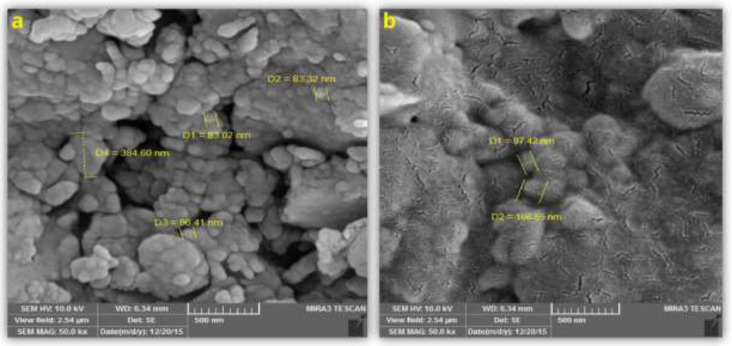
*SEM images of (a) NGRWR and* *(b) NGRWR-E*_8_* nanoparticles*

**Figure 2:**
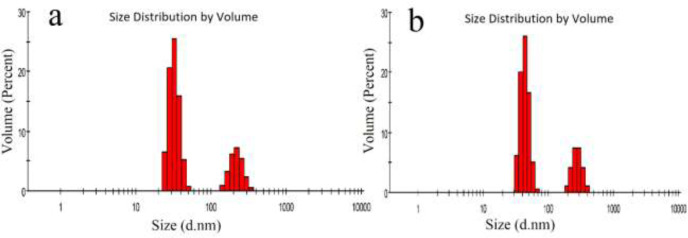
*Particle size distribution of* *(a) NGRWR peptide and (b)* *NGRWR-E*_8_* nanoparticles*

The CPPs were synthesized by the SPS method on CTC resin in a stepwise manner. Table 1 summarizes the properties of obtained CPPs as well as E_8_. W_4_R_4_-MTX possessed the highest loading efficiency (97%) and drug to peptide percentage (24.02%). The lowest loading efficiency (36%) and drug to peptide percentage (8.76%) was seen for NGRWK-MTX conjugates. SEM was applied to evaluate the size and morphology of NGRWR peptide and NGRWR-E_8_ nanoparticles. Figure 1 indicates the spherical morphology of NGRWR peptide with a size of ~100 nm. On the other hand, the salt bridge between peptide sequence and E_8_ resulted in the rod-like structure of NGRWR-E_8_ nanoparticles with not much change in particle size compared to the free peptide. Based on results obtained from Zetasizer (Figure 2), the size distribution of NGRWR peptide was measured to be 26 nm (43%), 37 nm (20%), 50 nm (16%), and 50-250 nm (21%) with a zeta potential of +11 mv.

Furthermore, the size distribution of NGRWR-E_8_ nanoparticles was in the range of 28-280 nm, where 45% of particles were below 50 nm with a zeta potential of +6 mV. The integration of E_8_ in nanoparticles is the main reason for their lower surface charge compared to peptide alone. The relatively high surface charge in both samples and the resulted electrostatic repulsion justify their good solution stability. 

*Cell viability assay*

**Figure 3:**
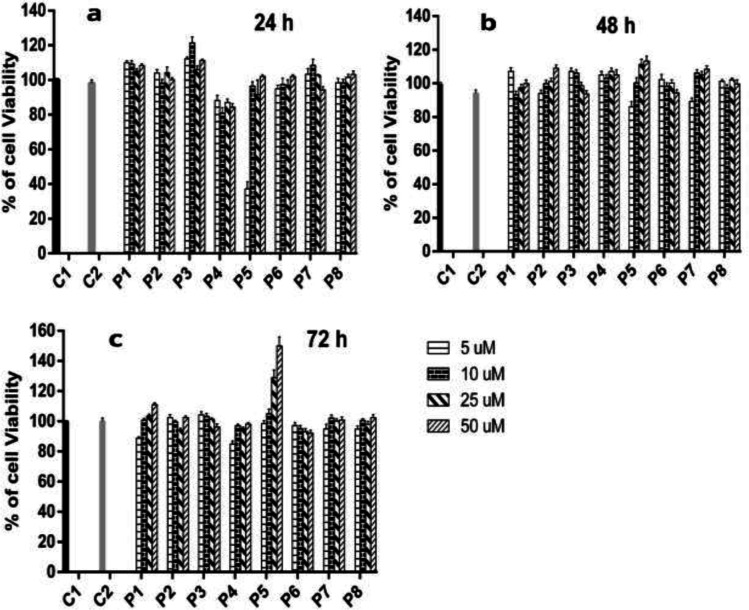
*Cell viability rates of different CPPs. Raji cells were incubated with CPPs for (a)* *24 h, (b) 48 h, and (c) 72 h at concentrations of 5, 10, 25, and 50 μM (a). The values represent the mean of at least three independent experiments performed in duplicate. (C1: Control group, C2: Control-DMSO (0.2%), P1: W4K4, P2: NGRWR, P3: WRNGRWR, P4: W4R4, P5: [WR]4, P6: R6, P7: WRNGR, P8: [WK]4).*

MTT assay was employed to evaluate the cytotoxic behavior of different CPPs and peptide-MTX conjugates in Raji cells. Based on results, cells treated with W_4_K_4_, W_4_R_4_, [WK]_4_, WRNGRWR, WRNGR, NGRWR, and R_6 _peptides exhibited no significant cytotoxicity (*p* 0.05) at concentrations of 5, 10, 25, and 50 μM after 24, 48, and 72 h (Figure 3). However, 5 μM of [WR]_4 _reduced the cell viability down to 40% after 24 h (Figure 3a). Moreover, cells were treated with peptide-MTX conjugates to establish the cytotoxic profile of peptide-drug conjugations for future experiments. 

**Figure 4:**
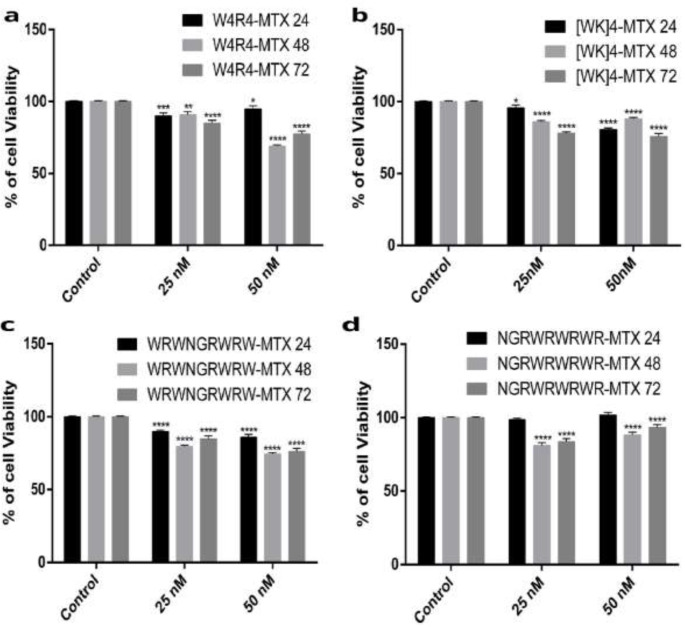
Cell viability rates of W4R4-MTX (4a), [WK]4-MTX (4b), WRNGRWR-MTX (4c) and NGRWR-MTX (4d) after 24, 48 and 72 hr at concentrations of 25 and 50 nM of MTX. ^*^P < 0.05, ^**^P < 0.01, ^***^P < 0.001, ^****^P < 0.0001

It was shown that 48 h after treatments with drug complexes of W_4_R_4_, W_4_K_4_, [WK]_4_, WRNGRWR, WRNGR, and NGRWR, there was no significant cytotoxic effect at a concentration of 25 nM MTX (Figure 4). As there was a time-dependent change in cell viability of [WR]_4_-MTX, NGRWK-MTX and R_6_-MTX, thus we removed them from the following experiments. Therefore, W_4_R_4_, W_4_K_4_, [WK]_4_, WRNGRWR, WRNGR, and NGRWR peptides and their conjugations with MTX were the only candidates for the following *in-vitro* immunogenic experiments.

*In-vitro immunogenicity assay*

**Figure 5:**
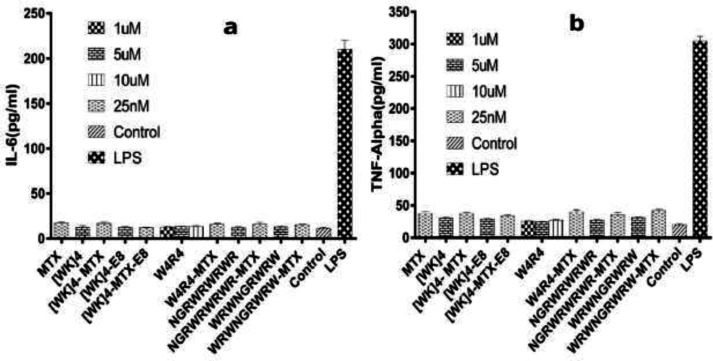
Cytokine release amount (pg/mL) in Raji cells was measured by ELISA assay. IL-6 (a) and TNF-α (b) release in cell culture supernatants were measured after 48 h of incubation with four peptides, their conjugations with MTX, [WK]4-E_8_ nanoparticles, [WK]4-MTX-E_8_ nanoparticles, and MTX. LPS (10 μg/mL) was used as a positive control, and untreated well was used as the negative control. The values represent the mean of at least three independent experiments performed in duplicate (mean ± SEM) decrease in viability was considered significant at *P* ≤ 0.05

ELISA test was applied to investigate the immunogenicity of CPPs and peptide-MTX conjugates in the Raji cell line. We observed no significant effect on IL-6 (Figure 5a) or TNF-*α* (Figure 5b) release profile of cells treated with CPPs either alone or in conjugation with MTX (*P* > 0.05). A modest increase in cytokine activity triggered by W_4_R_4_-MTX and WRNGRWR-MTX was not considered statistically significant. Indicated influence on the release of IL-6 and TNF-*α* was insignificant compared to LPS (as a positive control for Raji cells) after 48 h of incubation.

**Table 1:** The peptide sequences and properties

**CPPs**	**Amino acid sequences**	**Charge at pH 7.3**	**Molecular weight (g/mole)**	**MTX loading to CPPs**
W_4_K_4_	WWWWKKKK	+4	1275.54	%93
W_4_R_4_	WWWWRRRR	+4	1378.62	%97
[WK]_4_	WKWKWKWK	+4	1275.54	%87
[WR]_4_	WRWRWRWR	+4	1378.62	%88
WRNGRWR	WRWNGRWRW	+3	1416.61	%95
WRNGR	WRWRWRNGR	+4	1386.59	%72
NGRWR	NGRWRWRWR	+4	1386.59	%73
R_6_	RRRRRR	+6	955.19	%42
E_8_	EEEEEEEE	-8	1050.94	-

## Discussion

Nanocarriers are rapidly growing towards modifying several restrictions of conventional drug delivery systems for cancer therapy (12, 16). Peptide transporters are among the most well-known nano-drug delivery systems, which firstly was described in the 1988s and later became known as CPPs (1). CPPs offer exciting potential for effective delivery of biological molecules to the cytoplasm, which is otherwise impermeable to the cell membrane (30). However, their unwanted cytotoxic effects can limit clinical exposure to these nanocarriers. This study was performed to evaluate the cytotoxic and immunogenic behavior of several synthetic CPPs on the CPP-mediated transfer of MTX *in-vitro*.

Even though CPPs are presented as short sequences with no significant effect on the biological system, numerous studies have been done examining the biological impacts of various CPPs, including cationic and amphipathic ones (21). Amongst which, amphipathic sequences have demonstrated more toxicity on cell metabolism compared to cationic ones. For instance, as an amphipathic peptide, TP10 was found to affect cellular metabolism more than cationic Penetratin, and TAT (31). However, it has been shown that TP10 and its chemically modified derivatives induced no significant cytotoxicity and immunotoxicity at the concentrations of 10 μM and 5 μM *in-vitro* and 5 mg/kg of animal *in-vivo* (32). In previous reports, several amphipathic peptides were successfully applied to traverse the cell membrane with no significant cytotoxicity (1, 33). There have been other reports on the cytotoxic impacts of amphipathic peptides that proposed novel synthetic peptides as novel drug carriers for chemotherapeutics with the least cytotoxicity *in-vitro*. According to another study, there was a time- and dose-dependent toxicity with synthetic amphipathic peptides comprising tryptophan and arginine residues (34). Another group examined the *in-vitro* immunotoxicity of HIV-TAT, antennapedia, and transportan in A549 (alveolar), A431 (epidermal), and Caco-2 (intestinal) epithelial cells after 72 h. Consequently, there was no significant enhancement detected in inducing inflammatory cytokines in epithelial cells, indicating that these commonly used CPPs are passive carriers (34). Similarly, a cationic polyarginine peptide and penetration, at the dose of 1000 equivalent to their IC_50_, exhibited no toxic effect. However, it has been reported that TAT peptide could affect cell metabolism and cause some mild swelling in the rabbit’s cornea after 7 days (14, 31 and 32). Although the recent cytotoxic and immunogenic evaluations of different peptide sequences have been indicated no significant cell metabolism induction and biological responses, comprehensive investigations yet to be carried out. On the other hand, several advanced technologies such as nano-vectors (polymeric and liposomal), electroporation, genomics, and proteomics have been applied to overcome the challenges associated with cancer treatments (34, 35). Furthermore, the application of CPPs to achieve an efficient delivery system is critical in the field of chemotherapy. MTX is a folate antagonist that has been used for the treatment of various malignancies (36). The dose-dependent side effects of standard chemotherapeutics like MTX made them unsuitable for long-term therapies. It is essential to explore the potential applications of CPPs to deliver the therapeutic dosage of drugs to the cytoplasm of desired cells. The present study has addressed a significant safety concern regarding the application of CPPs for the efficient delivery of these drugs. In the present study, we evaluated the possible cytotoxic and immunogenic behavior of a new range of novel peptide sequences and their drug conjugates to elicit their possible role in the triggering of immune subsets or biological responses. Putting all together, we did not observe any significant cell viability reduction in Raji cells treated with each type of CPPs, as well as no activation of cytokine release after 48 h incubation with selected CPPs and peptide-MTX conjugates. Up to 50 μM, peptides illustrated no cytotoxic effect on Raji cells, while there was an exception for [WR]_4_ at 5µM after 24 h (Figure 3). Moreover, the conjugation of NGRWK, R_6_, and [WR]_4_ with MTX presented intensive cytotoxicity by reducing the cell viability down to 40%. Therefore, we removed cytotoxic peptide-MTX conjugates in the further immunogenic assessment. None of the measured cytokines (IL-*6* or TNF-*α*) were overexpressed in the Raji cells after incubation with selected CPPs, peptide-MTX conjugates, or nanoparticles. Meanwhile, as expected, cells incubated with LPS as positive control illustrated cytokine level enhancement 100-times higher than the control group (Figure 5). 

## Conclusion

In summary, we have demonstrated that the W_4_R_4_, W_4_K_4_, [WK]_4_, WRNGRWR, WRNGR, and NGRWR peptides and their conjugations with MTX have no cytotoxic and immunogenic effects at estimated concentrations. The obtained evidence supports the potential of designed CPPs as promising carriers and holds a significant agreement for their safe applications. However, long-term immunogenicity and *in-vivo* cytotoxicity investigations must be carried out to confirm the data.
